# Strong Optomechanical Coupling in Nanobeam Cavities based on Hetero Optomechanical Crystals

**DOI:** 10.1038/srep15964

**Published:** 2015-11-04

**Authors:** Zhilei Huang, Kaiyu Cui, Yongzhuo Li, Xue Feng, Fang Liu, Wei Zhang, Yidong Huang

**Affiliations:** 1 Department of Electronic Engineering, Tsinghua National Laboratory for Information Science and Technology, Tsinghua University, Beijing, 100084, China

## Abstract

Nanobeam cavities based on hetero optomechanical crystals are proposed. With optical and mechanical modes separately confined by two types of periodic structures, the mechanical frequency is designed as high as 5.88 GHz. Due to the optical field and the strain field concentrated in the optomechanical cavity and resembling each other with an enhanced overlap, a high optomechanical coupling rate of 1.31 MHz is predicted.

A trend for the next generation of optoelectronic devices is to introduce various particles and quasiparticle, including phonon, exciton, etc., as well as the coupling effect with photons, into the devices. These fundamental particles and quasiparticles form coupled systems with photons and may form the so-called polaritons^1^, through energy and momentum exchanges. The energy-momentum relations of the polaritons can be considered as general energy-bands, in analogy to the energy-bands of electrons. General energy-band engineering can similarly manipulate the polaritons to realize functional devices.

Cavity optomechanics explores the interaction between light and mechanical motion, and has attracted tremendous interest for its prospect to study quantum phenomena of mesoscopic mechanical objects^2^,^3^. Additionally, it is promising for many technological applications including sensing^4^, transducing^5^,^6^, and optical isolating^7^. Thanks to the rapid development of nanofabrication technology, cavity optomechanical systems have been realized in several types of systems, including vibrating microtoroids^3^, stacked microdisks^8^, and optomechanical crystals^9^ or, also referred to as phoxonic crystals^4^,^10^,^11^,^12^,^13^, which acts as both photonic crystals and phononic crystals. Among these systems, optomechanical crystals have attracted great interest because their relatively high mechanical frequency^2^ makes the mechanical vibration resistant to the thermal fluctuation^14^ and high optomechanical coupling rate^15^,^16^ results in strong interaction strength between photons and phonons in the cavities. Based on optomechanical crystals, cooling mechanical motion to the ground state^2^, coherent optical wavelength conversion^17^, and high precision accelerometer^18^ have been demonstrated. Using optomechanical crystals to realize quantum memories and networks^19^,^20^, co-guidance of slow optical and acoustic wave^13^, and biochemical sensing^4^ have also been proposed. However, further increment of the mechanical frequency and optomechanical coupling rate is hindered by the conventional optomechanical-crystal designs, in which the confinement of both mechanical and optical modes is achieved by the same periodical structure^9^, and the localized optical and mechanical modes are within the photonic and phononic bandgaps simultaneously formed by one periodic structure.

In this work, nanobeam cavities based on hetero optomechanical crystals with two types of periodic structures are proposed to break this constrain. With the optical and mechanical modes separately confined by two periodic structures, the confinement of the mechanical mode can be designed independent with the confinement of the optical mode. Consequently, a mechanical frequency as high as 5.88 GHz is designed while the working wavelength of the optical cavity is maintained at 1.55 μm. Due to the design flexibility in the hetero structure, the optical field and the strain field are designed to be concentrated inside the optomechanical cavities and resemble each other with an enhanced overlap. After optimization, a record high optomechanical coupling rate of 1.31 MHz is predicted.

## Results

### Proposed structure

**Figure 1:**
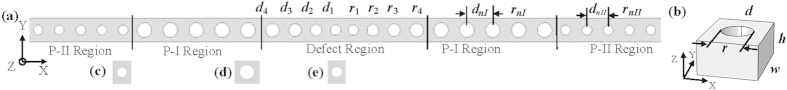
(**a**) The plan-view schematic of the proposed optomechanical crystal nanobeam cavities. (**b**) The unit cell constituting the structure. (**c–e**) The unit cells constituting P-II, P-I regions and one of the two central holes.

The plan-view schematic of the proposed hetero optomechanical crystal nanobeam cavities is shown in Fig. 1(a). The material used is silicon-on-insulator (SOI). The gradient air-holes at the centre of the structure with different radii (*r*_*i*_) and separation distances (*d*_*i*_) constitute the defect region, which is sandwiched by two types of periodic structures, Periodic-I (P-I) and Periodic-II (P-II) regions. A unit cell of this nanobeam is shown in Fig. 1(b), with a uniform width (*w*) of 455 nm and a height (*h*) of 220 nm. The radii of the holes in P-I and P-II region (*r*_*n*I_, *r*_*n*II_) are 136 nm and 90 nm, respectively. The pitches in P-I and P-II region are 487 nm and 360 nm, respectively. The radii of the gradient holes (*r*_1_−*r*_4_) and the corresponding pitches vary linearly from 99 nm to 127 nm and from 368 nm to 458 nm, respectively. Here, the linearity of the variation in the defect region is adopted because good optical properties have previously been demonstrated in this linear gradient nanobeam structure^21^. The plan view of the unit cells of P-II, P-I region, and one of the two central holes are shown in Fig. 1(c–e).

### High- frequency mechanical mode confined by the hetero structure

To analyse how the defect modes are confined in this structure, the optical and mechanical bands of the unit cells in P-I, P-II, and the defect region are calculated. Here, all the band structures are calculated based on the unit cell with Bloch boundary condition, i.e., effective band structures of the infinite structures reduplicated by the unit cell in the *x* direction is computed.

**Figure 2:**
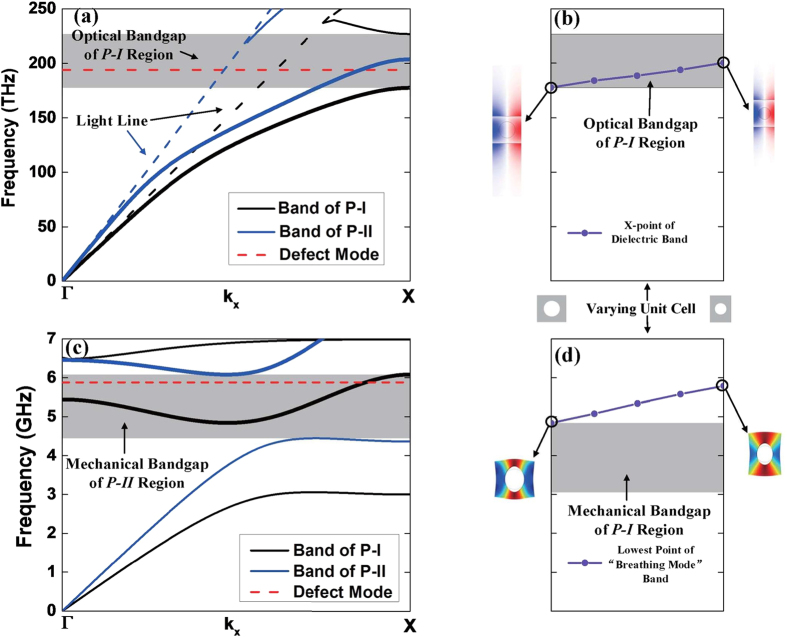
(**a**) The optical band of the periodic region. The red dash line represents the cavity optical mode at the frequency of 194 THz. (**b**) The frequency variation of the X point of dielectric bands as the unit cell transforming from Fig. 1(d–e). The corresponding mode profiles of these two unit cells are shown in the bottom. The grey areas in (**a**,**b**) represent the optical bandgap formed by P-I region. (**c**) The *y*-, *z*-symmetric mechanical band of the periodic region. The red dash line represents the mechanical defect mode at the frequency of 5.88 GHz. (d) The frequency variation of the lowest point of the breathing-mode bands as the unit cell transforming from Fig. 1(d–e). The corresponding mode profiles of these two unit cells are shown in the bottom. The grey areas in (**c,d**) represent the mechanical bandgap formed by P-II and P-I region, respectively.

Due to the different geometric parameters, P-I and P-II region possess different optical and mechanical bands and bandgaps. Figure 2(a) shows the optical bands of P-I and P-II region, where the leaky modes above the light line are omitted. It should be noted that the wave vector of X-point represents different values in the two kinds of periodic structures so that the light lines do not overlap. The thick lines indicate the dielectric bands in Fig. 2(a). Figure 2(c) shows the mechanical bands of *y*-, *z*-symmetric modes, where the thick lines indicate the “breathing-mode”^9^ bands. Here, the breathing-modes are the resonant mechanical modes of nanobeam cavities. The grey areas represent the optical or mechanical bandgap in Fig. 2.

In order to clarify the function of the hetero structure, the changes for the optical mode and mechanical mode under gradient structure are calculated and analysed. Figure 2(b,d) respectively present the frequency variations of the dielectric optical band and the lowest point of the mechanical “breathing-mode” band at the X-point, while the radii and pitches of the air holes of the unit cell shrink from the side to the centre, i.e., from Fig. 1(d) in P-I region to (e) in the defect region. As shown in Fig. 2(b), the optical frequency of the dielectric mode increases and goes into the bandgap formed by P-I region as reducing the radii and pitches of the air holes of the unit cell, which corresponds to the structural transformation from side to centre of the nanobeam. When the optical frequency of the dielectric mode lies in the bandgap, the optical mode can be well confined. Thus the optical mode of the defect unit cell of Fig. 1(e) is reflected by the side mirror-region, which forms an optical resonator^21^,^22^. For mechanical mode, the structure transformation with reducing the radii and pitches of the air holes in the defect region is capable of increasing the frequency of the mechanical mode, as calculated in Fig. 2(d), and helpful for obtaining a higher mechanical frequency. However, this structure transformation also pushes the mechanical frequency of “breathing mode” out of the bandgap formed by P-I region, as shown in Fig. 2(d). It means that only with P-I region, the optical field can be confined well, but the acoustic wave will leak to the waveguide. Thus, further increment of the mechanical frequency is hindered by the conventional optomechanical-crystal designs, in which the confinement of both mechanical and optical modes is achieved by the same periodical structure, and the localized optical and mechanical modes are within the photonic and phononic bandgaps simultaneously formed by one periodic structure. In this work, nanobeam cavities based on hetero optomechanical crystals with two types of periodic structures are proposed to break this constrain. With the optical and mechanical modes separately confined by two periodic structures, the confinement of the mechanical mode can be designed independently with the confinement of the optical mode.

Based on this principle, P-II region with smaller air radius and pitch is added to confine the mechanical defect mode within mechanical bandgap in a higher frequency range, as shown in Fig. 2(c). As a result, the defect optical and mechanical mode are separately confined by P-I and P-II region.

**Figure 3:**
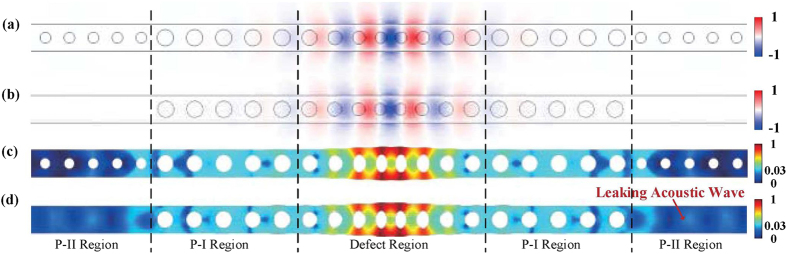
(**a**) The electric *y*-component (*E*_*y*_) of the optical defect mode of the whole structure and (**b**) that of the structure without P-II region. (**c**) The magnitude of the displacement field (**q**) of the mechanical mode of the whole structure and (**d**) that of the structure without P-II region. The acoustic wave leak to the waveguide in structure without P-II region. The colour bar of the displacement filed is set to be nonlinear to clearly display the leaky acoustic wave.

The modes of the structure are calculated and presented in the red dash lines in Fig. 2 (a,c). The mode profiles for the optical and mechanical modes are shown in Fig. 3. The frequency of the optical mode is 194 THz (corresponding to a wavelength of 1.55 μm) and the optical Q-factor is 2.0 × 10^4^, which is mainly limited by radiation loss. Both of the frequency and the Q-factor of the optical mode are invariant to the existence of P-II region as the optical mode is almost completely confined within P-I region. The situation is different for the mechanical mode. Without P-II region, the mechanical frequency is 5.88 GHz, which exceeds the range of the bandgap formed by P-I region and leaks to the waveguide as shown in Fig. 3(d). Since the amplitude of the acoustic wave leaking to the waveguides is much smaller than the maximum field in the defect region, the colour legends of Fig. 3(c,d) are set to be nonlinear to show the leaking part of the acoustic wave clearly. Considering the acoustic leak losses and thermoelastic effect^23^ at the environmental temperature of 20 K, the mechanical Q-factor is calculated to be 1.0 × 10^3^, dominantly limited by the leakage. While, by adding P-II region, the mechanical frequency is invariant but mechanical Q-factor of the whole structure increases to 4.9 × 10^5^, indicating the defect mechanical mode is well confined.

Unlike the conventional optomechanical crystal designed with one period, the proposed hetero structure here can separately confine the optical and mechanical modes by two types of periodic structures. More importantly, the defect mechanical mode with frequency too high to be confined by the bandgap of P-I region can be confined by P-II region. Thus, the hetero nanobeam is capable of confining higher mechanical frequency mode than other conventional nanobeams. The concept and the method developed in this work can be generally applied to the design of 2D optomechanical crystals as well. Moreover, the geometric parameters can be designed with more flexibility in the hetero structures so that strong optomechanical coupling can be maximized by further optimizing the structure.

### Strong coupling between mechanical and optical modes

We next focus on achieving a high optomechanical coupling rate (*g*) in this proposed structure. For an optomechanical cavity, the optomechanical coupling rate represents the interaction strength between the optical and the mechanical mode. Quantitatively, the coupling rate is the optical frequency shift caused by zero-point motion of mechanical mode as shown in Eq. (1)^6^


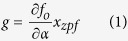


In this equation, *f*_o_ is the frequency of the optical mode, *α* is the normalized mechanical vibration amplitude, and *x*_zpf_ is the vibration amplitude of zero-point fluctuation. For a dielectric optomechanical cavity, two effects contribute to the frequency shift^16^, the photoelastic effect^24^ and the moving dielectric boundary effect^6^. Given by Chan *et al.*^16^, Eq. (2) and (3) calculate the optomechanical coupling rate due to the photoelastic effect and the moving dielectric boundary effect, respectively,


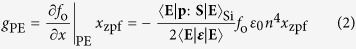



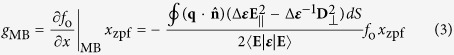


where **p** is the photoelastic tensor of silicon, **S** is the strain tensor of the mechanical mode, *n* is the refractive index of silicon, **nˆ** is the surface normal vector, **q** is the normalized displacement field, **E** is the electric field, **D** is the electric displacement filed, and the subscripts 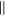 and 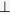 denote the parallel and perpendicular components to the surface, respectively. Here, the strain tensor (**S**) is the differential of displacement field, i.e. *S*_*ij*_  = 1*/*2(d*q*_*i*_*/*d*x*_*j*_ +  d*q*_*j*_ */*d*x*_*i*_).

We mainly study the enhancement of the photoelastic effect since it is the dominant effect for a typical nanobeam optomechanical crystal^16^. By investigating Eq. (2), we find that the contribution of photoelastic effect is proportional to the overlap integral of electric field of optical mode and strain field of mechanical mode. Thus, the strain field is more essential than the displacement field when we consider the optomechanical coupling rate in the nanobeam structure. To enhance the optomechanical coupling strength, making the optical field and the strain field confined in the optomechanical cavities and resemble each other with an enhanced overlap is proposed in this work.

**Figure 4:**
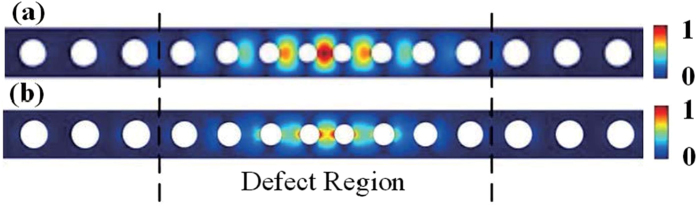
(**a**) The absolute value of electric *y*-component (|*E*_*y*|)_ of the optical mode. (**b**) The absolute value of strain *yy*-component (|*S*_*yy*|)_ of the mechanical mode. For the sake of clarity, only the fields in the central part are shown, since the fields outside the central part are negligibly small.

Figure 4(a,b) show the absolute value of *y*-component of electric field and *yy*-component of strain field, the dominant component of each field, and their coupling as given in Eq. (2). To further concentrate the electric field and the strain field in the same region to enhance the overlap integral, the defect region of the proposed structure is designed by two principles. First, the radii and the pitches of the air holes are reduced from side to centre. Because both fields mainly concentrate in the silicon region, reducing the radii and the pitches of the air holes makes their interaction stronger. Although by doing so, the mechanical frequency increases and exceeds the upper frequency of the mechanical bandgap formed by P-I region, the mechanical mode can be well confined in the whole hetero structure. Second, an even number, instead of an odd number, of air holes distribute symmetrically in the defect to ensure the centre of the cavity is occupied by silicon. Thus, the maxima of the electric and strain fields only exist at the central region of the nanobeam. Based on these two principles, we design the air holes in defect region with both radius and pitch shrinking linearly, as shown in Fig. 1. Therefore, six geometric parameters determine the structure without P-II region, i.e. the width of the nanobeam, the radius and pitch of air holes in P-I region, the shrinking rate of radius and pitch and the pitch between the two centre holes. With the width determined by maintaining the optical mode at 194 THz, the other five parameters are optimized using Nelder-Mead method^26^, a downhill simplex optimization method, to achieve the maximum coupling rate. (See the method section for more details.) Therefore, the structure is optimized and determined based on the maximum of the optomechanical coupling rate. However, for this type of structure with shrinking central holes, the mechanical frequency of them exceeds the frequency range that can be confined by P-I region inevitably, as shown in Fig. 2(d) and Fig. 3(d). So we use P-II region to confine the corresponding mechanical mode, as analysed in the above section. It should be noted that although the optimization process for enhancing the coupling does not refer to P-II region, P-II region can confine a mechanical mode with higher frequency, which has a strong coupling with the optical mode and supports the optimised coupling in this way. It is found that, at the maximum point of the optomechanical coupling rate, the optical field and the strain field are concentrated in the cavity central region and resemble each other with an enhanced overlap, so that the optomechanical coupling rate is enhanced by the optimization. Based on our calculation, the optomechanical coupling rate of the optimized structure contributed by photoelastic effect is as high as 1.03 MHz. With in-phase contribution of moving dielectric boundary effect of 0.28 MHz, the total optomechanical coupling rate of the cavity is as high as 1.31 MHz. Table 1 shows the mechanical frequency (*f*_m_) and theoretical predicted optomechanical coupling rate (*g*) of nanobeam optomechanical structures based on SOI chip. Compared with other reported results, the mechanical frequency for the proposed hetero structure is relatively high. In addition, due to the design flexibility in the hetero structure, the optical field and the strain field are designed to be concentrated inside the optomechanical cavities and resemble each other with an enhanced overlap. After optimization, a record high optomechanical coupling rate of 1.31 MHz is predicted. Strong optomechanical coupling is important for most applications of optomechanical systems.

## Discussion

In conclusion, we have proposed nanobeam cavities based on hetero optomechanical crystals. We find that using two types of periodic structures, the optical mode and the mechanical mode can be separately confined by two bandgaps. Accordingly, a high mechanical resonant frequency of 5.88 GHz is achievable in the proposed structure. Additionally, the geometric parameters can be further optimized. After optimization, the optical field and the strain field are both confined in the optomechanical cavity and their mode profiles resemble each other with an enhanced overlap. Consequently, an optomechanical coupling rate up to 1.31 MHz is achievable, which is the highest optomechanical coupling rate reported to the best of our knowledge. The concept and the method developed in this work can be generally applied to the design of 2D optomechanical crystals as well.

## Methods

The optical band structures in this paper are calculated by plane wave expansion^27^ (PWE) method. Other simulations, including the mechanical bands and the optical and mechanical modes, are achieved by finite element method (FEM). In the mechanical band calculation, the two facets perpendicular to the *x* direction of the unit cell is set with Bloch boundary condition. Other facets are set with free boundary condition as no constrains acting on these facets.

In the calculation of optical modes, the designed structure is surrounded with large air domain, so that the evanescent field is damped enough at the boundaries, where scattering boundary condition is set. Absorption domains are set at two sides of the beam when calculated the mechanical modes, to get leakage portion of the acoustic wave.

In the optical simulation, the refractive index of silicon is set to be 3.48. In the mechanical simulation, the density of the silicon is set to be 2300 kg/m^3^ and full anisotropic elasticity matrix is used, where the three independent component (*c*_11_, *c*_12_, *c*_44_) = (165.5, 63.9, 79.5) GPa^28^.

For calculating the optomechanical coupling rate, the optical and mechanical modes are simultaneously simulated in the same mesh system, so that the domain and surface integration as shown in Eq. (2) and (3) can be computed. Here, the photoelastic tensor (*p*_11_, *p*_12_, *p*_44_) is set to be (−0.094, 0.017, −0.051)^24^.

The specific geometric parameters are determined by using Nelder-Mead optimization method^26^, a downhill simplex method. The structure can be determined by 6 geometric parameters, i.e. the width of the nanobeam, the radius and pitch of air holes in P-I region, the shrinking rate of radius and pitch and the pitch between the two centre holes. During the optimization, one parameter, the width, is determined to tune the optical mode resonating at 194 THz (corresponding to the wavelength of 1.55 μm). Then, the other 5 parameters, forming a 5-demensional space, are structure parameters to be optimized. That is to say, each point in the 5-demensional space stands for a specific structure and the corresponding optomechanical coupling rate (*g*) of it can be calculated as mentioned above. Since optimization process usually search minimum, we set the objective function to be -*g* to achieve the largest optomechanical coupling rate. For each round of optimization, 6 points in the 5-demensional space are randomly generated as initial values, which form a high dimensional triangle. After iteratively reflect, expand, contract or reducing, the triangle converge to a local minimum point of the objective function. Thus, with 30 rounds of optimization, we can find the largest optomechanical coupling rate and the corresponding structure parameters.

## Additional Information

**How to cite this article**: Huang, Z. *et al.* Strong Optomechanical Coupling in Nanobeam Cavities based on Hetero Optomechanical Crystals. *Sci. Rep.* **5**, 15964; doi: 10.1038/srep15964 (2015).

## Figures and Tables

**Table 1:** Comparison of mechanical frequency (*f*_m_) and theoretical predicted (with experimental measured result denoted with *) optomechanical coupling rate (*g*) of nanobeam optomechanical structures based on SOI chip.

Description of the structure	*f*_m_ (GHz)	*g* (MHz)
Nanobeam with rectangular holes^9^	2.2	0.22
Nanobeam with oval hole^16^	5.1	0.86 (1.10*)
Corrugated nanobeam^29^	4.0	0.54
This work	5.9	1.31

Optical frequency (*f*_o_) is used in this work to calculate *g* as shown in Eq. (2) and (3), which equivalents to *g*/2π when optical angular frequency (ω_o_) is used.
